# Cerebral lesion correlates of sympathetic cardiovascular activation in multiple sclerosis

**DOI:** 10.1002/hbm.24759

**Published:** 2019-08-12

**Authors:** Klemens Winder, Ralf A. Linker, Frank Seifert, Ruihao Wang, De‐Hyung Lee, Tobias Engelhorn, Arnd Dörfler, Kilian Fröhlich, Max Hilz

**Affiliations:** ^1^ Department of Neurology Friedrich‐Alexander‐Universität Erlangen‐Nürnberg Erlangen Germany; ^2^ Department of Neurology University Regensburg Regensburg Germany; ^3^ Department of Neuroradiology Friedrich‐Alexander‐Universität Erlangen‐Nürnberg Erlangen Germany; ^4^ Department of Neurology Icahn School of Medicine at Mount Sinai New York New York

**Keywords:** autonomic dysfunction, insular lesion, multiple sclerosis, visceral arousal, voxel‐based lesion symptom mapping

## Abstract

Cardiovascular autonomic dysfunction is common in multiple sclerosis (MS) and contributes significantly to disability. We hypothesized that cerebral MS‐lesions in specific areas of the central autonomic network might account for imbalance of the sympathetic and parasympathetic cardiovascular modulation. Therefore, we used voxel‐based lesion symptom mapping (VLSM) to determine associations between cardiovascular autonomic dysfunction and cerebral MS‐related lesion sites. In 74 MS‐patients (mean age 37.0 ± 10.5 years), we recorded electrocardiographic RR‐intervals and systolic and diastolic blood pressure. Using trigonometric regressive spectral analysis, we assessed low (0.04–0.15 Hz) and high (0.15–0.5 Hz) frequency RR‐interval‐and blood pressure‐oscillations and determined parasympathetically mediated RR‐interval–high‐frequency modulation, mainly sympathetically mediated RR‐interval–low‐frequency modulation, sympathetically mediated blood pressure‐low‐frequency modulation, and the ratios of sympathetic and parasympathetic RR‐interval‐modulation as an index of sympathetic‐parasympathetic balance. Cerebral MS‐lesions were analyzed on imaging scans. We performed a VLSM‐analysis correlating parameters of autonomic dysfunction with cerebral MS‐lesion sites. The VLSM‐analysis showed associations between increased RR‐interval low‐frequency/high‐frequency ratios and lesions most prominently in the left insular, hippocampal, and right frontal inferior opercular region, and a smaller lesion cluster in the right middle cerebellar peduncle. Increased blood pressure‐low‐frequency powers were associated with lesions primarily in the right posterior parietal white matter and again left insular region. Our data indicate associations between a shift of cardiovascular sympathetic‐parasympathetic balance toward increased sympathetic modulation and left insular and hippocampal lesions, areas of the central autonomic network. The VLSM‐analysis further distinguished between right inferior fronto‐opercular lesions disinhibiting cardiac sympathetic activation and right posterior parietal lesions increasing sympathetic blood pressure modulation.

## 1 INTRODUCTION

Multiple sclerosis (MS) is frequently associated with autonomic dysfunction which afflicts up to 84% of MS‐patients and may comprise cardiovascular dysregulation, bowel, bladder, and sexual dysfunction, or sudomotor abnormalities (Hilz, 2016; Kaplan, Berkowitz, & Samuels, 2015; Merkelbach et al., 2006; Racosta, Kimpinski, Morrow, & Kremenchutzky, 2015; Winder et al., 2016). Particularly, cardiovascular dysfunction is associated with an increased risk of complications and reduced life expectancy (Hilz, 2016; Kaplan et al., 2015; Midaglia et al., 2016), and may manifest as baroreflex dysfunction with orthostatic intolerance or orthostatic hypotension, postural tachycardia syndrome, and altered sympathetic or parasympathetic heart rate modulation (Hilz, 2016; Kaplan et al., 2015; Merkelbach et al., 2006; Racosta et al., 2015).

Autonomic cardiovascular modulation depends on the integrity of the central autonomic network which includes the insular and cingulate cortices, the amygdala, the prefrontal or orbitofrontal cortex, the hypothalamus, and brainstem structures (Benarroch, 1997; Racosta et al., 2015; Winder et al., 2017). MS‐associated lesions in the periventricular white matter, juxtacortical U‐fibers, the brainstem, the cerebellum or spinal cord areas (Ge, 2006; Vigeveno, Wiebenga, Wattjes, Geurts, & Barkhof, 2012; Winder et al., 2016) might compromise the centrally mediated sympathetic and parasympathetic cardiovascular modulation (Hilz, 2016; Racosta et al., 2015).

While previous, region of interest‐based studies (Acevedo, Nava, Arriada, Violante, & Corona, 2000; Saari et al., 2004; Vita et al., 1993) associated cardiovascular autonomic dysregulation in MS‐patients either with their total lesion load and lesions in the midbrain and parietal white matter (Saari et al., 2004) or with pontine lesions (Acevedo et al., 2000; Vita et al., 1993), we hypothesize that neuro‐inflammatory lesions close to or within specific supratentorial master controllers of the autonomic nervous system account for an imbalance of the sympathetic and parasympathetic cardiovascular modulation.

To identify neuro‐inflammatory lesion sites associated with cardiovascular autonomic dysregulation, we assessed the lesion load throughout the whole brain using magnetic resonance imaging (MRI) and applied voxel‐based lesion symptom mapping (VLSM) to correlate the location of lesions with changes in the sympathetic and parasympathetic balance of cardiovascular modulation in MS‐patients (Bates et al., 2003; Rorden, Karnath, & Bonilha, 2007; Winder et al., 2015, 2016).

## 2 MATERIALS AND METHODS

### 2.1 Patients

Anonymized data used in this study are available on request from Dr. Winder. Among outpatients seen between November 2012 and October 2014 at the Multiple Sclerosis Clinic at the University Hospital Erlangen of the Friedrich‐Alexander Universität Erlangen‐Nürnberg, we screened 90 patients (62 females; mean age 37.2 ± 10.3 years) who had been diagnosed with relapsing remitting MS according to the 2010 revised McDonald criteria (Polman et al., 2011) and who had been recommended for treatment with fingolimod (Hilz et al., 2017). Since all patients were about to receive immunomodulatory therapy with fingolimod, patients who had received previous disease modifying treatments were taken off their previous medication for at least the period consistent with current recommendations (Pelletier & Hafler, 2012). This assured that we only included patients in the current study who were on no immunomodulatory therapy that might have affected autonomic cardiovascular assessment (Hilz et al., 2017). For our voxel‐wise analysis, we studied MS‐patients with the following inclusion criteria: (a) age 18–65 years, (b) MRI scans of good quality including T1, T2, or proton‐density (PD), and fluid‐attenuated inversion recovery (FLAIR) sequences available for lesion analysis and spatial normalization. We excluded patients with other structural diseases of the central nervous system, such as stroke or microangiopathic lesions, as well as patients with diseases or therapies that might alter autonomic nervous system function (Hilz et al., 2017). We took the medical history with particular emphasis on disease course, autonomic symptoms, co‐morbidities and medication, and performed a physical examination. The degree of physical disability was rated using the Expanded Disability Status Scale (EDSS) scores ranging from 0 to 9 (Kurtzke, 1983). The study was approved by the Ethics Committee of the Friedrich‐Alexander Universität Erlangen‐Nürnberg, Germany, and registered at the German Clinical Trial Register (DRKS00004548). Prior to the study, all patients had given their written informed consent according to the declaration of Helsinki.

### 2.2 Assessment of autonomic cardiovascular parameters

Autonomic cardiovascular parameters of our patients were tested between 9 a.m. and 4 p.m., after a resting period of at least 40 min to ensure a stable cardiovascular situation (Hilz et al., 2017). We assessed cardiovascular autonomic function in a quiet room with an ambient temperature of 24°C and stable humidity. We recorded RR‐intervals (RRIs) (ms) by 3‐lead electrocardiography, systolic blood pressure (BPsys; mmHg), and diastolic blood pressure (BPdia; mmHg) by finger‐pulse photoplethysmography (Portapres; TPD‐Biomedical Instrumentation, Amsterdam, the Netherlands; Hilz et al., 2015, 2017). From each of the 3 min recordings, we extracted the most stationary and artifact‐free 120 s epochs to average values of RRIs, BPsys, and BPdia to calculate further autonomic parameters described below (Hilz et al., 2017). Biosignal data were digitized and displayed on a personal computer and a custom designed data acquisition and analysis system (SUEmpathy™, SUESS‐Medizintechnik, Germany) and stored for offline analysis (Hilz et al., 2017).

To assess cardiovascular sympathetic and parasympathetic modulation in the frequency domain, we performed trigonometric regressive spectral analyses (TRSs) of RRI and BPsys values sampled during the 120 s epochs (Hilz et al., 2017). We determined sympathetic and parasympathetic modulation of RRI and BP in the low‐frequency (LF; 0.04–0.14 Hz) and high‐frequency (HF; 0.15–0.50 Hz) ranges (Hilz et al., 2010, 2011, 2017). LF oscillations of RRI at rest reflect sympathetic outflow and, to an undetermined degree, also parasympathetic modulation; LF oscillations of BP represent sympathetic outflow only. HF oscillations of RRIs reflect cardiac parasympathetic modulation (Goldstein, Bentho, Park, & Sharabi, 2011; Hilz et al., 2017; Rahman, Pechnik, Gross, Sewell, & Goldstein, 2011), whereas BP fluctuations in the HF range are primarily a mechanical consequence of respiration‐induced fluctuations in venous return and cardiac output (Hilz et al., 2017). The magnitude of LF and HF oscillations was determined as the integral under the power spectral density curves of RRI (ms^2^/Hz) and BP (mmHg^2^/Hz) for the LF and HF frequency bands, and was expressed as LF and HF powers of RRI (ms^2^) and BP (mmHg^2^; Hilz et al., 2017). As measure of the balance between sympathetic and parasympathetic influences on heart rate modulation, we calculated the ratio between RRI oscillations in the LF and HF ranges, that is, the LF/HF‐ratios of RRI (Hilz et al., 2017).

### 2.3 MR imaging of the brain

All patients underwent brain imaging using 1.5 Tesla (Siemens Magnetom Aera) or 3 Tesla (Siemens Magnetom Trio) MRI. The MRI scans were performed according to a dedicated protocol including axial native T1, T2, or PD, diffusion‐weighted imaging, as well as axial and coronal T1 sequences after injection of intravenous Gadolinium‐based contrast media. FLAIR sequences were acquired directly in the axial and sagittal plane or as a 3D‐FLAIR with reconstruction in the axial, coronal and sagittal plane.

### 2.4 Lesion analysis and spatial normalization

**Figure 1:**
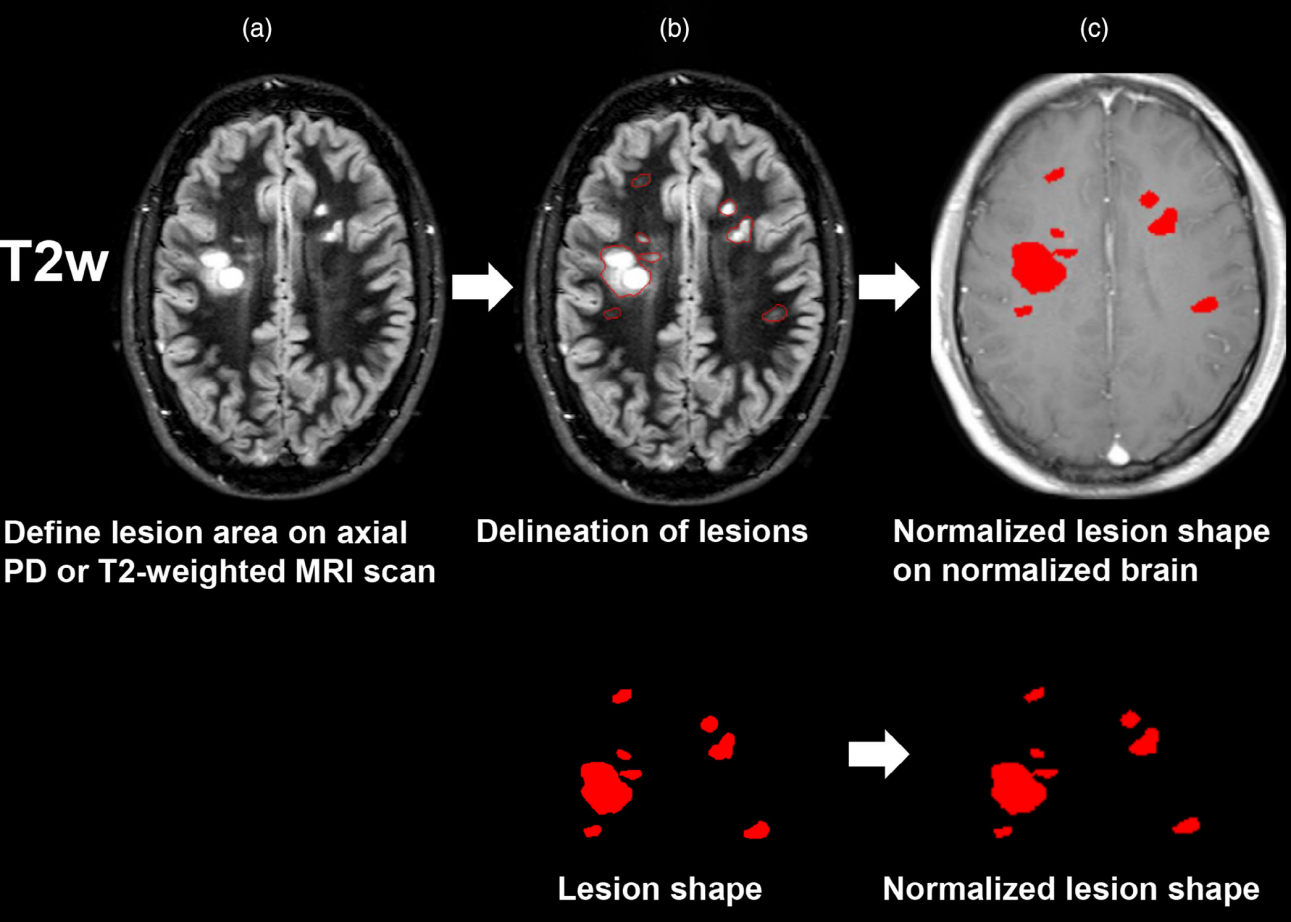
Process of lesion delineation and spatial normalization. (a) T2‐weighted MR images in original space. (b) The T2‐weighted axial image was used to define the area of T2‐hyperintense MS‐lesions shown as a red contour and below the red lesion shape. (c) The lesion shape, the T2‐weighted as well as the T1‐weighted MRI scans were then transformed into stereotaxic space which generated the normalized map. The normalized lesion map was also applied to the normalized T1‐weighted brain to qualitatively demonstrate the accuracy of image normalization [Color figure can be viewed at http://wileyonlinelibrary.com]

Two experienced investigators (K.W. and F.S.) manually delineated the boundaries of the MS‐lesions on anonymized axial T2‐weighted MRI scans using MRIcron (http://www.mccauslandcenter.sc.edu/mricro/mricron/; Rorden, Bonilha, Fridriksson, Bender, & Karnath, 2012). To ensure that no perivascular spaces were scored as MS‐lesions, lesions were only delineated if they were detectable as a hyperintense signal on T2 as well as FLAIR scans (Winder et al., 2016). To avoid observer bias, both raters were blinded to clinical and autonomic parameters during imaging analysis. The MRI scan and the lesion shape were transferred into stereotaxic space using the normalization algorithm of SPM12 (http://www.fil.ion.ucl.ac.uk/spm/) and the Clinical Toolbox for SPM12 (Rorden et al., 2012) (http://www.mricro.com/clinical-toolbox/spm8-scripts). We applied the MR‐segment‐normalize algorithm of the Clinical Toolbox to transform the MRI‐derived lesion shape and the MR images to the standardized T1 template based on younger individuals with a resampled voxel size of 1 × 1 × 1 mm^3^ (Rorden et al., 2012). The process of lesion analysis and delineation as well as spatial normalization is illustrated in Figure 1. The normalized lesion map was analyzed with the latest version of nonparametric mapping (NPM)‐software implemented in the MRIcron software package (Rorden et al., 2012).

### 2.5 Statistical analysis

As a first step, we determined the lesion overlap by overlaying lesion shapes of all MS‐patients who were eligible to be included in the VLSM‐analysis. Then, we compared parameters of cardiovascular function voxel‐by‐voxel between dichotomous overlap values (lesion or no lesion in a given voxel) of MS‐lesion sites, identified in the VLSM‐analysis, with the patients' continuous values of autonomic parameters using *t*‐test statistics (Rorden et al., 2007). Only voxels that were lesioned in at least four patients were included in the voxel‐wise analysis. To control for multiple comparisons, we applied a false discovery rate (FDR) correction of *q* < 0.01. Since increasing total cerebral lesion volume might be associated with an increased risk of afflicting brain areas that are strategically relevant for autonomic function (Karnath, Fruhmann Berger, Küker, & Rorden, 2004; Winder et al., 2015, 2017), we calculated the total volume of MS‐lesions throughout the brain using the latest version NPM software implemented in the MRIcron software package (Rorden et al., 2007). To determine damaged brain regions, affected voxels were overlaid on the automated anatomical labeling (AAL) atlas (Tzourio‐Mazoyer et al., 2002) or the John Hopkins University (JHU)‐White matter‐labels atlas (1 mm). The peak coordinates of the involved regions are presented in Montreal Neurological Institute (MNI) space. To graduate the strength of associations between autonomic cardiovascular parameters and the lesion site, we analyzed *z*‐scores of cortical (AAL atlas) and white matter (JHu‐atlas) lesions.

To further determine parameters possibly contributing to autonomic dysfunction, we correlated parameters of autonomic cardiovascular function that showed significant voxel‐wise associations with patient age, disease duration, EDSS scores, and total cerebral lesion volume using the Spearman rank correlation coefficient. To test for normal distribution of data, we used the Shapiro–Wilk test. Normally distributed data are presented as mean ± standard deviation (SD) and non‐normally distributed data as median and interquartile ranges (IQRs). Statistical significance was assumed for *p* < .05. For statistical calculations, we used a commercially available statistic program (SPSS 20.0; IBM, Armonk, NY).

## 3 RESULTS

### 3.1 Patient characteristics

Of the 90 MS‐patients screened, 74 patients (54 women and 20 men) fulfilled the inclusion criteria and were therefore included in the VLSM‐analysis. Demographic and clinical data as well as Spearman rank correlation coefficients between autonomic parameters associated with cerebral lesion sites and age, disease duration, EDSS scores, and lesion volume are shown in Table 1. Fifty‐five of the 74 patients (74.3%) had 1.5 T MRIs while 19 patients (25.7%) had 3 T MRIs. None of the patients presented with any overt clinical signs of cardiovascular autonomic dysfunction. Mean RRI was 789.4 ± 88.6 ms, mean heart rate was 76.9 ± 8.6/min. Median BPsys was 114.6 mmHg (IQR 105.2–125.9 mmHg) and median BPdia was 62.3 mmHg (IQR 53.5–69.0 mmHg).

**Table 1:** Demographic and clinical parameters of the multiple sclerosis patients as well as correlations with frequency domain parameters that showed significant correlations with cerebral lesion sites

			Correlation with RRI‐LF/HF‐ratio		Correlation with LF‐BPsys‐power	
Variable	Median	IQR	Spearman rho	*p*	Spearman rho	*P*
Patient age, year	36.5	28.8–46.5	0.05	.70	−0.03	.80
Disease duration (months)	65.6	25.7–120.5	0.15	.20	0.13	.29
EDSS score	2.3	1.5–3.5	0.05	.70	−0.09	.45
Lesion volume (ml)	15.6	4.2–37.5	0.20	.10	0.15	.21

Abbreviations: EDSS, expanded disability status scale; IQR, interquartile range; LF‐BPsys‐power, systolic blood pressure‐low‐frequency powers; RRI‐LF/HF, ratio of RR‐interval low‐frequency/high‐frequency oscillations.

### 3.2 Autonomic frequency domain parameters

Median RRI‐LF‐power was 657.3 ms^2^ (IQR = 321.5 ms^2^‐1,396.1 ms^2^), median RRI‐HF‐power was 235.5 ms^2^ (IQR = 119.8 ms^2^‐533.7 ms^2^), and median RRI‐LF/HF‐ratio was 3.3 (IQR = 1.5–6.1). Median BPsys‐LF‐power was 11.5 mmHg^2^ (IQR = 7.3 mmHg^2^–20.3 mmHg^2^). RRI‐LF/HF‐ratios, the parameter of cardiac sympathetic and parasympathetic balance, and sympathetically mediated BPsys‐LF‐powers showed a significant association with cerebral MS‐lesion sites. Neither RRI‐LF/HF‐ratios nor BPsys‐LF‐powers correlated with patient age, disease duration, EDSS scores, and total cerebral lesion volume (Table 1).

### 3.3 Voxel‐based lesion symptom mapping

**Figure 2:**
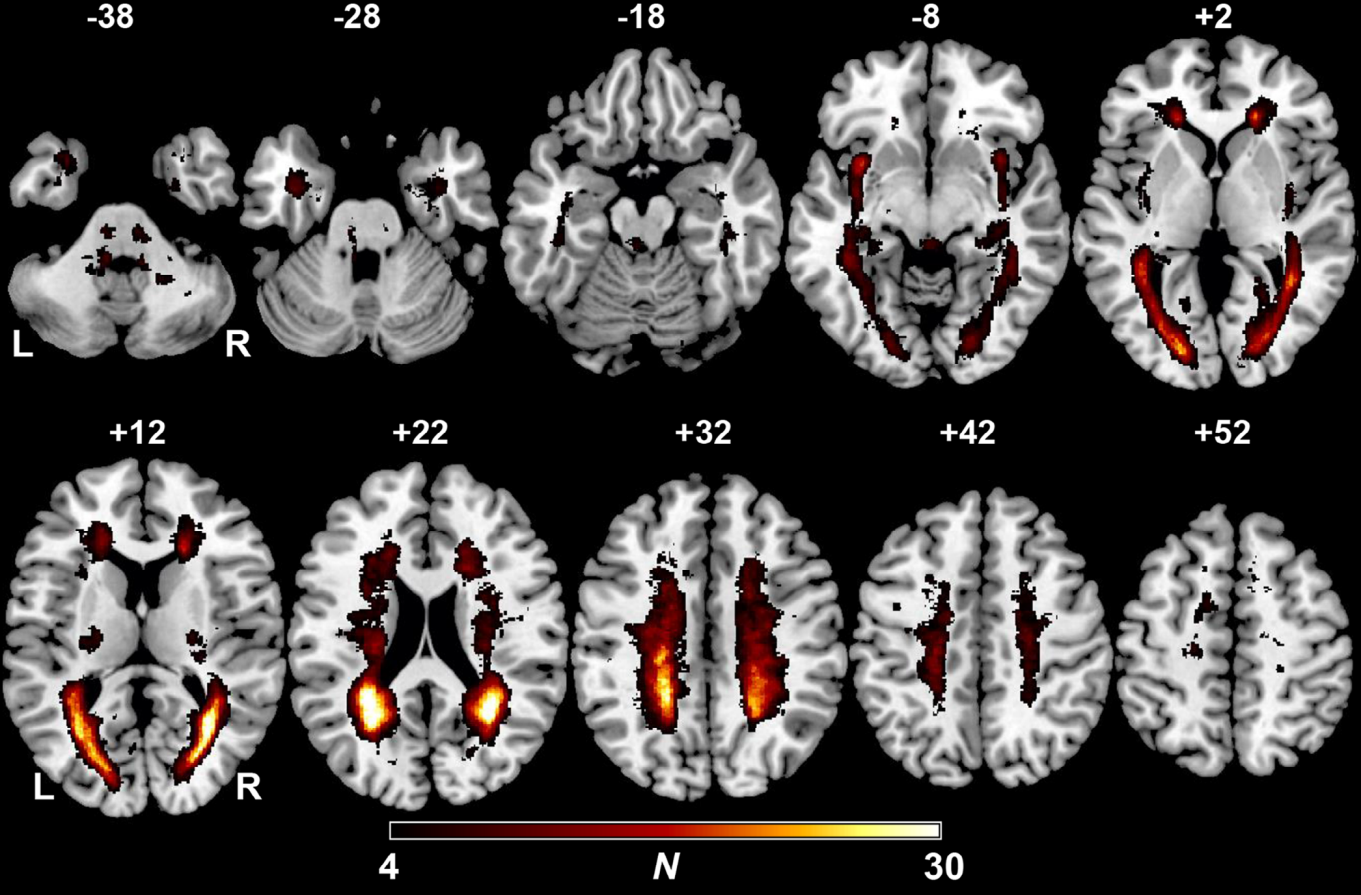
Overlap and distribution of T2‐hyperintense multiple sclerosis lesions of all patients. The number of overlapping lesions is illustrated by different colors coding increasing frequencies from dark red to yellow. Regions with higher lesion overlap counts are found symmetrically in periventricular regions, most prominently in the parietal periventricular white matter, as well as in the subinsular regions, and periaqueductal midbrain gray. Montreal Neurological Institute (MNI) *z*‐coordinates of each transverse section are given. L, left hemisphere; *N*, number of individuals with a lesion in a given voxel; R, right hemisphere [Color figure can be viewed at http://wileyonlinelibrary.com]

**Figure 3:**
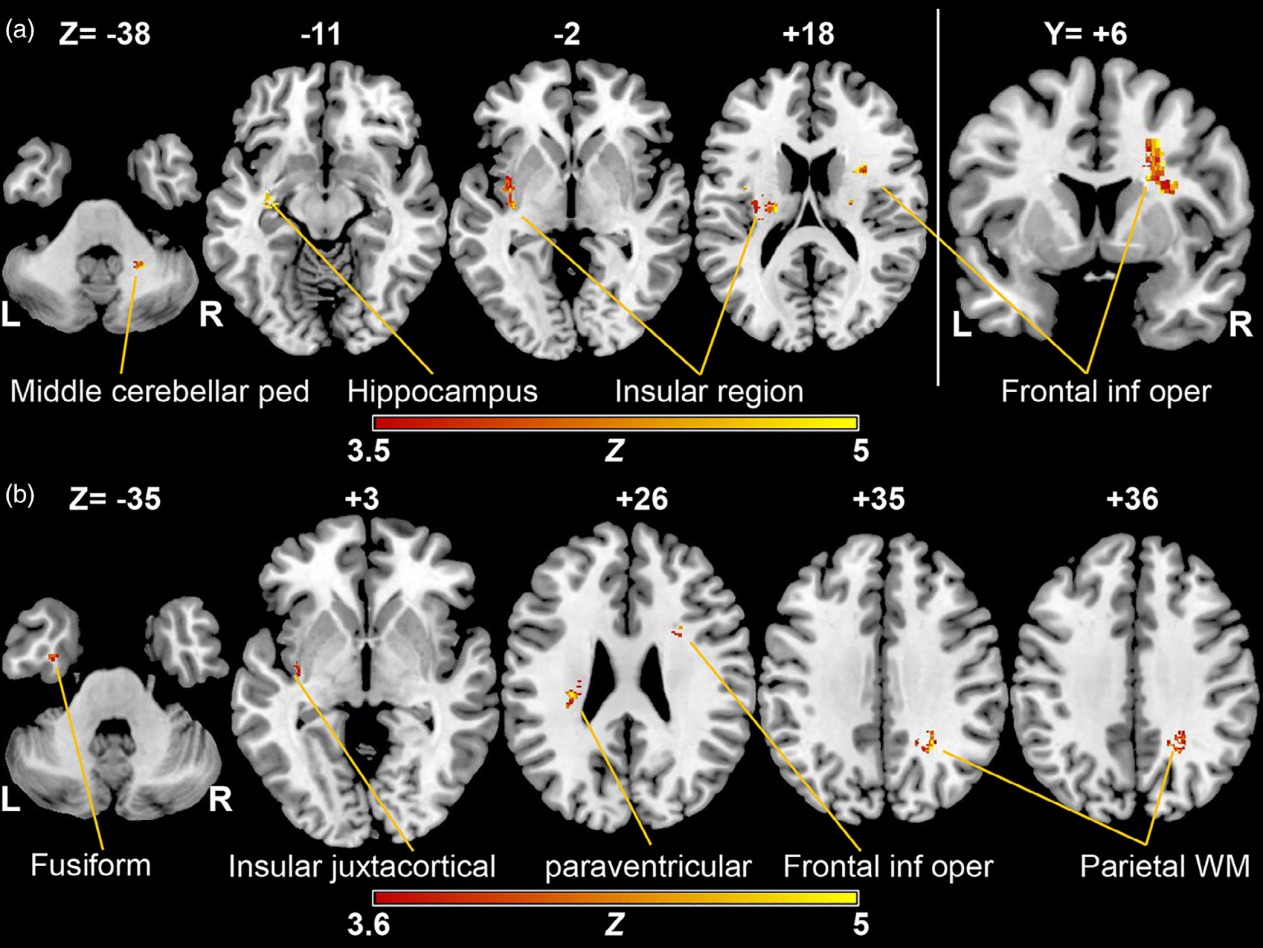
Results of the voxel‐wise *t*‐test statistics comparing parameters of autonomic arousal between patients with and without lesions in a given voxel. Lesioned voxels in the left insular juxtacortical white matter, hippocampus (axial slices), and right inferior opercular juxtacortical region (coronal slice) were most prominently associated with a shift of cardiac sympatho‐vagal balance toward increased sympathetic modulation, as evidenced by increased LF/HF‐ratios of RR‐intervals. A false discovery rate (FDR) correction of *q* < 0.01 was applied (*z*‐score = 3.5). A smaller lesion cluster correlated also in the right middle cerebellar peduncle (a). Lesioned voxels most prominently in the right posterior parietal‐juxtacortical region, as well as in the left insular regions were associated with increased sympathetic blood pressure modulation, as shown by an increase of BPsys‐LF‐powers (*z*‐score = 3.6). Only voxels that were damaged in at least four individuals were included in the analysis. L, left hemisphere; R, right hemisphere; *z* = *z*‐score [Color figure can be viewed at http://wileyonlinelibrary.com]

Figure 2 shows the lesion distribution and overlap‐map of lesioned voxels in all patients thresholded to include only voxels that were lesioned in at least four individuals. As mentioned above, RRI‐LF/HF‐ratios and BPsys‐LF‐powers correlated significantly with cerebral MS‐lesion sites: results of the voxel‐wise *t*‐test statistics comparing the RRI‐LF/HF‐ratios between patients with and without lesions in a given voxel are shown in Table 2 and Figure 3a. A total of 2,978 lesioned voxels correlated with increasing RRI‐LF/HF‐ratios. Four hundred voxels (13.4%) were located in the gray matter and 2,578 voxels (86.6%) in the white matter.

**Table 2:** Result of the voxel‐based lesion symptom mapping analysis of RR‐interval LF/HF‐ratios using voxel‐wise *t*‐test statistics

Areas with lesioned voxels associated with increasing RR‐interval LF/HF‐ratios (as determined by voxel‐wise *t*‐testing^a^)	Number of lesioned voxels	Peak coordinates of lesion sites		
		*x*	*y*	*z*
*Lesions in AAL areas*				
Right frontal inferior operculum	9	29	10	31
Left rolandic operculum	6	−38	−4	17
Left insula	190	−38	−4	18
Right insula	11	37	−4	6
Left hippocampus	51	−34	−16	−11
Left caudate	45	−20	−19	23
Right caudate	12	21	5	23
Left putamen	40	−33	−16	−5
Right putamen	7	36	−5	3
Left thalamus	3	−21	−18	15
Left superior temporal gyrus	1	−40	−7	−6
Right cerebellum 8	25	21	−52	−42
*Lesions in white matter tracts*				
Right middle cerebellar peduncle	103	24	−51	−40
Left posterior limb of internal capsule	157	−25	−14	15
Right posterior limb of internal capsule	78	23	−14	8
Right anterior corona radiata	1	26	13	24
Left superior corona radiata	424	−26	−14	19
Right superior corona radiata	370	26	9	24
Left posterior corona radiata	19	−29	−24	21
Left external capsule	357	−33	−8	−9
Right external capsule	2	25	5	18
Fornix	20	−34	−16	−11
Left superior longitudinal fasciculus	172	−34	−6	24
Right superior longitudinal fasciculus	132	31	3	19
Right superior fronto‐occipital	4	21	5	23

*Note*: *x*, *y*, and *z* indicate the peak coordinates of corresponding voxel counts outlined in Montreal Neurological Institute space. AAL, automated anatomical labeling.

a Voxel‐wise *t*‐tests comparing RR‐interval LF/HF‐ratios of patients with MS‐lesions with RR‐interval LF/HF‐ratios of patients without MS‐lesions in a given voxel.

In short, higher RRI‐LF/HF‐ratios were associated with lesioned voxels most prominently in the left insular region, hippocampus, and paraventricular white matter extending into the left superior insular region, and associated with juxtacortical lesions in the inferior‐frontal opercular sites. A small cluster of lesioned voxels in the right middle cerebellar peduncle also correlated with increased RRI‐LF/HF‐ratios. Table 2 shows lesion sites associated with increased RRI‐LF/HF‐ratios, the corresponding number of damaged voxels, and peak coordinates outlined in MNI space. The highest *z*‐score predicting increased RRI‐LF/HF‐ratios in AAL areas was 7.0 (left caudate) followed by the right inferior opercular region (*z*‐score = 5.2). The highest *z*‐score predicting increased RRI‐LF/HF‐ratios in white matter areas was 6.5 (left posterior corona radiata, suprainsular region).

Results of the voxel‐wise *t*‐test statistics comparing the BPsys‐LF‐powers between patients with and without lesions in a given voxel are shown in Table 3 and Figure 3b. A total of 912 voxels correlated with increased BPsys‐LF‐powers, 191 (20.9%) were located in the gray matter and 721 voxels (79.1%) were in the white matter. Increased BPsys‐LF‐powers correlated with lesion sites most prominently in the right posterior parietal white matter, left juxtacortical insular region and adjacent external capsule, left and right corona radiata. The highest *z*‐score in AAL areas, that is, most predictive lesion site for increasing BPsys‐LF was 4.7 (left precuneus and cuneus). The highest z‐score in white matter areas predicting increased BPsys‐LF values was 6.5 (left posterior corona radiata, left suprainsular region), followed by 5.3 (right subcortical parietal white matter). Thus, caudate nucleus and white matter lesions seem to be more predictive of increasing RRI‐LF/HF‐ratios and BPsys‐LF values than cortical lesions, respectively.

**Table 3:** Result of the voxel‐based lesion symptom mapping analysis of BPsys‐LF‐powers using voxel‐wise *t*‐test statistics

Areas with lesioned voxels associated with increased BPsys‐LF‐powers (as determined by voxel‐wise *t*‐testing^a^)	Number of lesioned voxels	Peak coordinates of lesion sites		
		*x*	*y*	*z*
*Lesions in AAL areas*				
Right frontal inferior operculum	5	29	11	29
Left insula	13	−36	−13	2
Left hippocampus	6	−35	−12	−11
Right calcarine	14	21	−58	20
Left cuneus	9	−19	−59	28
Right cuneus	8	21	−58	21
Left superior occipital gyrus	24	−22	−61	27
Left fusiform	61	−34	−5	−37
Left precuneus	2	−19	−59	32
Right precuneus	39	22	−58	24
Left putamen	2	−35	−15	−6
Left inferior temporal gyrus	8	−34	−5	−35
*Lesions in white matter tracts*				
Splenium of corpus callosum	29	19	−52	20
Left superior corona radiate	156	−30	−23	30
Right superior corona radiate	30	24	10	24
Left posterior corona radiata	124	−32	−24	27
Right posterior corona radiate	3	19	−48	35
Left external capsule	75	−36	−13	−5
Left superior longitudinal fasciculus	2	−33	−24	29
Right superior longitudinal fasciculus	2	29	7	23

*Note*: x, y, and z indicate the peak coordinates of corresponding voxel counts outlined in Montreal Neurological Institute space. AAL, automated anatomical labeling.

a Voxel‐wise *t*‐tests comparing BPsys‐LF‐powers of patients with MS‐lesions with BPsys‐LF‐powers of patients without MS‐lesions in a given voxel.

## 4 DISCUSSION

In our MS‐patients, the VLSM‐analysis unveiled two major findings, associations between increased sympathetic cardiovascular modulation and specific sites of MS‐lesions: Increased RRI‐LF/HF‐ratios––indicting a shift of sympathetic‐parasympathetic cardiac modulation toward sympathetic predominance––were associated with T2‐weighted lesions most prominently in the left insular and hippocampal regions, callosal radiation as well as right inferior fronto‐opercular region, and also with a smaller lesion cluster in the right middle cerebellar peduncle (Figure 3a). Moreover, increased sympathetic blood pressure modulation, that is, increased sympathetically mediated BPsys‐LF‐powers were associated with T2‐weighted lesions, most prominently in the right posterior parietal‐juxtacortical region, and again in the above left insular region and callosal radiation (Figure 3b).

While autonomic dysfunction is common among MS‐patients and may contribute to disability and cardiovascular complications (Hilz, 2016; Kaplan et al., 2015; Merkelbach et al., 2006; Racosta et al., 2015), studies assessing the impact of isolated central lesions on cardiovascular dysregulation are scarce and show inconsistent results (Acevedo et al., 2000; Saari et al., 2004; Vita et al., 1993). Saari et al. (2004) found associations between decreased diastolic blood pressure responses to orthostatic challenge and the total intracranial lesion load, as well as the midbrain and parietal lesion volume. Acevedo et al. (2000) as well as Vita et al. (1993) observed associations between deteriorating cardiovascular autonomic function and brainstem lesions.

### 4.1 Voxel‐wise analyses show more precise correlation between autonomic dysfunction and lesion sites

Voxel‐wise analyses do not predefine lesion sites assumed to be associated with autonomic dysfunction but have the advantage of determining associations between the voxel‐wise lesion overlap in any brain area and parameters reflecting cardiovascular autonomic dysfunction (Winder et al., 2016, 2017).

While the voxel‐wise analyses showed the above associations between lesion sites and RRI‐LF/HF‐ratios as well as sympathetic BPsys‐LF‐powers, these two autonomic parameters neither correlated with parameters potentially confounding autonomic function, such as patient age, disease duration and severity, nor with the total volume of cerebral MS‐lesions. We therefore conclude that changes in cardiovascular autonomic function depend less on the overall lesion load but more on the site of neuro‐inflammatory lesions and their association with areas or pathways of the central autonomic network (Benarroch, 1997; Hilz et al., 2017; Winder et al., 2017).

### 4.2 Lesion sites associated with cardiovascular autonomic imbalance

Our findings confirm the relevance of the insular region for proper cardiovascular autonomic control (Cechetto & Shoemaker, 2009; Oppenheimer, 2006; Saper, 2002). In our patients, the increase in sympathetic cardiovascular modulation was associated with lesions in the insular and hippocampal region as well as paraventricular white matter with a predominance of left‐sided lesions confirming the lateralization of sympathetic and parasympathetic cardiovascular modulation (Hilz et al., 2001, 2006; Saper, 2002; Winder et al., 2015; Yoon, Morillo, Cechetto, & Hachinski, 1997; Zamrini et al., 1990). These regions are functionally closely connected to each other (Augustine, 1996). The insula integrates viscero‐autonomic signals and assures organotopic viscero‐sensory representation, and also generates autonomic arousal (Cechetto & Shoemaker, 2009; Critchley, Corfield, Chandler, Mathias, & Dolan, 2000; Oppenheimer, 2006; Saper, 2002; Sörös & Hachinski, 2012; Winder et al., 2015). Hemispheric inactivation, functional neuroimaging and electrical stimulation studies showed that the right insula contributes more to sympathetic modulation while the left insula is more involved in cardio‐vagal control (Critchley, Corfield, et al., 2000; Critchley, Elliott, Mathias, & Dolan, 2000; Hilz et al., 2001; Kimmerly, O'Leary, Menon, Gati, & Shoemaker, 2005; Oppenheimer, 2006; Oppenheimer, Gelb, Girvin, & Hachinski, 1992; Yoon et al., 1997; Zamrini et al., 1990). Animal experiments showed that insular cortex lesions trigger cardiovascular autonomic imbalance, which depends on the lesion side (Zhang, Rashba, & Oppenheimer, 1998). In patients with acute ischemic stroke involving the left insular region, Oppenheimer, Kedem, and Martin (1996) found an increase in cardiac sympathetic tone. Similarly, sympathetic heart rate and blood pressure modulation were increased in our MS‐patients with left insular lesions.

However, right‐sided neuro‐inflammatory lesions in regions surrounding the insular cortex were also associated with increased RRI‐LF/HF‐ratios and BPsys‐LF‐powers indicating augmented sympathetic cardiovascular modulation (Figure 3a,b). This finding supports the conclusions of Butcher and Cechetto that the right posterior insula has a sympatho‐inhibitory effect on the predominantly sympatho‐excitatory right‐hemispheric central autonomic network, in particular on the right anterior insular cortex (Butcher & Cechetto, 1995). In animal studies, Zhang et al. (1998) found that lesions in the right posterior insula disinhibit sympathetic modulation in rats. In humans, several studies confirmed this finding by showing associations between stroke‐induced right parietal and opercular lesions and sympatho‐excitatory effects with autonomic dysfunction, hyperglycemia, and myocardial damage (Ay et al., 2006; Winder et al., 2015).

Moreover, the VLSM‐analysis further differentiated effects of lesion sites on cardiac or blood pressure modulation. Our analysis showed that large neuro‐inflammatory lesion clusters in the right inferior fronto‐opercular region were associated with increased RRI‐LF/HF‐ratios indicating a shift of cardiac autonomic modulation toward enhanced sympathetic influence on the heart while large lesion clusters in the posterior parietal‐juxtacortical white matter were associated with increased BPsys‐LF‐powers indicating enhanced sympathetic blood pressure modulation (Figure 3, Table 3). These results support findings of insular cortex stimulation in animals (Oppenheimer & Cechetto, 1990; Yasui, Breder, Saper, & Cechetto, 1991) and humans (Oppenheimer et al., 1992) that showed different rostro‐caudal insular and juxtainsular representations of sympathetically mediated heart rate acceleration or slowing and of blood pressure increase or decrease. Oppenheimer et al. (1992) demonstrated in epilepsy patients that the right insula has an anterior–posterior organization of sympathetic blood pressure activation, a finding that supports our observation of increased sympathetically mediated blood pressure modulation in patients with neuro‐inflammatory lesions in the posterior parietal‐juxtacortical white matter.

Our data also show associations between increased RRI‐LF/HF‐ratios, that is, increased sympathetic heart rate modulation, and lesions in the right inferior frontal opercular region (Table 2, Figure 3a). In patients with right ventromedial prefrontal cortex lesions, Hilz et al. (2006) observed increased sympathetic cardiovascular activation in response to visual emotional stimulation. In healthy individuals, several functional imaging studies moreover showed involvement of the prefrontal cortex in physical or mental task‐induced sympathetic arousal (Critchley et al., 2005; Critchley, Corfield, et al., 2000; King, Menon, Hachinski, & Cechetto, 1999; Seifert et al., 2013). We therefore assume that the right inferior opercular region may have inhibitory effects on sympatho‐excitatory influences of the neighboring prefrontal cortex, and that the neuro‐inflammatory lesions within the right inferior frontal opercular region disinhibit sympatho‐excitatory effects within the adjacent prefrontal cortex.

Finally, our data showed associations between right cerebellar lesions and increased RRI‐LF/HF‐ratios, that is, increased sympathetic heart rate modulation. Again, functional imaging studies of cardiovascular sympathetic arousal have shown that cerebellar areas are involved in cardiovascular control and in cardiovascular responses (Critchley, Corfield, et al., 2000; Harper, Bandler, Spriggs, & Alger, 2000; King et al., 1999). Critchley, Corfield, et al. (2000) concluded that the cerebellum seems to integrate cardiovascular responses associated with cognitive or motor behavior, and seems to be involved in interoception of autonomic arousal states (Critchley, Wiens, Rotshtein, Ohman, & Dolan, 2004). We assume that neuro‐inflammatory right cerebellar lesions contribute to disinhibition of sympathetic cardiovascular control.

### 4.3 Limitations

There are several limitations to our study that require a careful interpretation of the results. The VLSM‐technique only supports conclusions regarding associations between autonomic cardiovascular imbalance and lesion sites for brain areas that are involved in the lesion overlap (Winder et al., 2016, 2017). Thus, associations between autonomic dysfunction and lesions in areas that usually contribute to autonomic processing, such as the orbitofrontal cortices or the hypothalamus, cannot be adequately determined by the VLSM approach unless the sample size is far bigger than in our study (Benarroch, 1997; Winder et al., 2016). However, our strict in‐ and exclusion criteria ruling out the evaluation of patients with other possible causes of autonomic dysfunction such as medication influencing the autonomic nervous system and metabolic or endocrine diseases with clinically overt signs of autonomic dysfunction limited the number of patients who were suited for this study. In addition to the MS‐lesion associated pathology, the anatomical vulnerability of the insula in terms of its distal vascularization might be relevant to its association with changes in cardiovascular control (Ture, Yasargil, Al‐Mefty, & Yasargil, 2000). Since MS‐lesions are mostly located in the juxtacortical or periventricular white matter but not primarily in the cortex itself (Ge, 2006; Vigeveno et al., 2012), our patients had the major lesion burden within white matter tracts and juxtacortical regions but not directly in the insular or fronto‐parietal cortex. However, white matter tracts interconnect central autonomic master control areas, such as the insular cortices, the cingulate gyrus, orbitofrontal area, or the amygdala of both hemispheres via the corona radiata and the corpus callosum (Augustine, 1996; Benarroch, 1997). In our patients, neuro‐inflammatory lesions adjacent but not within cortical autonomic structures such the insular cortex were, therefore, still associated with increased sympathetic cardiovascular modulation. Another limitation of our study results from the fact that the MS‐lesions were assessed on two different scanners, that is, 1.5 T MRIs in 55 of the 74 patients (74.3%) and with 3 T MRIs in 19 patients (25.7%). Uniform 3 T MRI might have refined our analysis and improved results of our VLSM‐analysis. Moreover, T2/FLAIR‐hyperintense lesions may reflect several different pathological substrates. We have to note that we studied autonomic parameters, more specifically frequency domain parameters at rest. Although our MS‐patients did not show overt symptoms of autonomic dysregulation, we cannot fully rule out they might have had subtle signs of autonomic dysfunction. We did not perform further autonomic challenge maneuvers to assess cardiovascular autonomic modulation, such as tilt table testing. Finally, we did not assess correlations between changes of autonomic function and MS‐lesions of the spinal cord since the VLSM‐method is not validated for lesion analyses within the spinal cord.

## 5 CONCLUSION

In conclusion, our VLSM‐data indicate associations between a shift of cardiovascular sympathetic‐parasympathetic balance toward increased sympathetic cardiovascular modulation and lesions in the left insular region and hippocampus, areas of the central autonomic network. The VLSM‐analysis further discriminated between right inferior fronto‐opercular lesions disinhibiting cardiac sympathetic activation and right posterior parietal lesions increasing sympathetic blood pressure modulation.

## CONFLICT OF INTERESTS

F.S. received travel or speaker honoraria from Merz Pharmaceuticals GmbH, funding for travel to conference from Novartis Pharma GmbH, speaker honoraria from Desitin Arzneimittel GmbH. M.J.H. received personal compensation for activities from Sanofi‐Genzyme, Germany and Novartis Pharma GmbH, Germany. D.H.L. and RAL received travel grants or speaker honoraria from Bayer Health Care Pharmaceuticals (NJ), Biogen Idec (Cambridge, MA), Merck Serono (Germany), Novartis Pharma GmbH, and TEVA Pharmaceutical Industries (Germany).

## ETHICAL APPROVAL

All procedures performed in studies involving human participants were in accordance with the ethical standards of the institutional and national research committee and with the 1964 Helsinki declaration and its later amendments or comparable ethical standards.

## INFORMED CONSENT

Informed consent was obtained from all individual participants included in the study.

## Data Availability

Anonymized data used in this study are available on request from Dr. Winder.
